# Enhancing Omics Research of Crop Responses to Drought under Field Conditions

**DOI:** 10.3389/fpls.2017.00174

**Published:** 2017-02-14

**Authors:** Si Wu, Fen Ning, Qinbin Zhang, Xiaolin Wu, Wei Wang

**Affiliations:** Collaborative Innovation Center of Henan Grain Crops, State Key Laboratory of Wheat and Maize Crop Science, College of Life Sciences, Henan Agricultural UniversityZhengzhou, China

**Keywords:** crop plants, drought treatment, field experiment, omics analysis, soil water deficit, stress response

## Many but limited useful data from OMICS analysis of crop responses to drought

Crop production relies heavily on rainfall during the growing season, especially in developing countries. Drought, due largely to the effects of soil water deficit, is the most serious abiotic stress limiting crop production, accounting for ~70% potential yield loss worldwide (Salekdeh et al., 2009). Therefore, a major goal for global agriculture is to develop drought-tolerant crops. To this end, a fully understanding of physiological, biochemical, and gene regulatory networks relating to drought tolerance in plants is essential (Valliyodan and Nguyen, 2006). In this aspect, tremendous advances have been made over the past decade. Particularly, morphological, physiological traits, and biochemical changes that are relevant for drought tolerance have been well documented (e.g., Feller and Vaseva, 2014; Lynch et al., 2014; Simova-Stoilova et al., 2016). Various omics approaches, including genomics, transcriptomics, proteomics, and metabolomics, as well as the combinations of them—systems biology, have been used to elucidate the complex mechanisms of drought stress responses in crops (Shanker et al., 2014; Budak et al., 2015). However, the systematic mechanism of drought responses in crops and its application in drought tolerance improvement remain largely unclear, because of limited systematic biology data available from field experiments and genotype × environment interaction in complex, often unknown ways.

**Figure 1:**
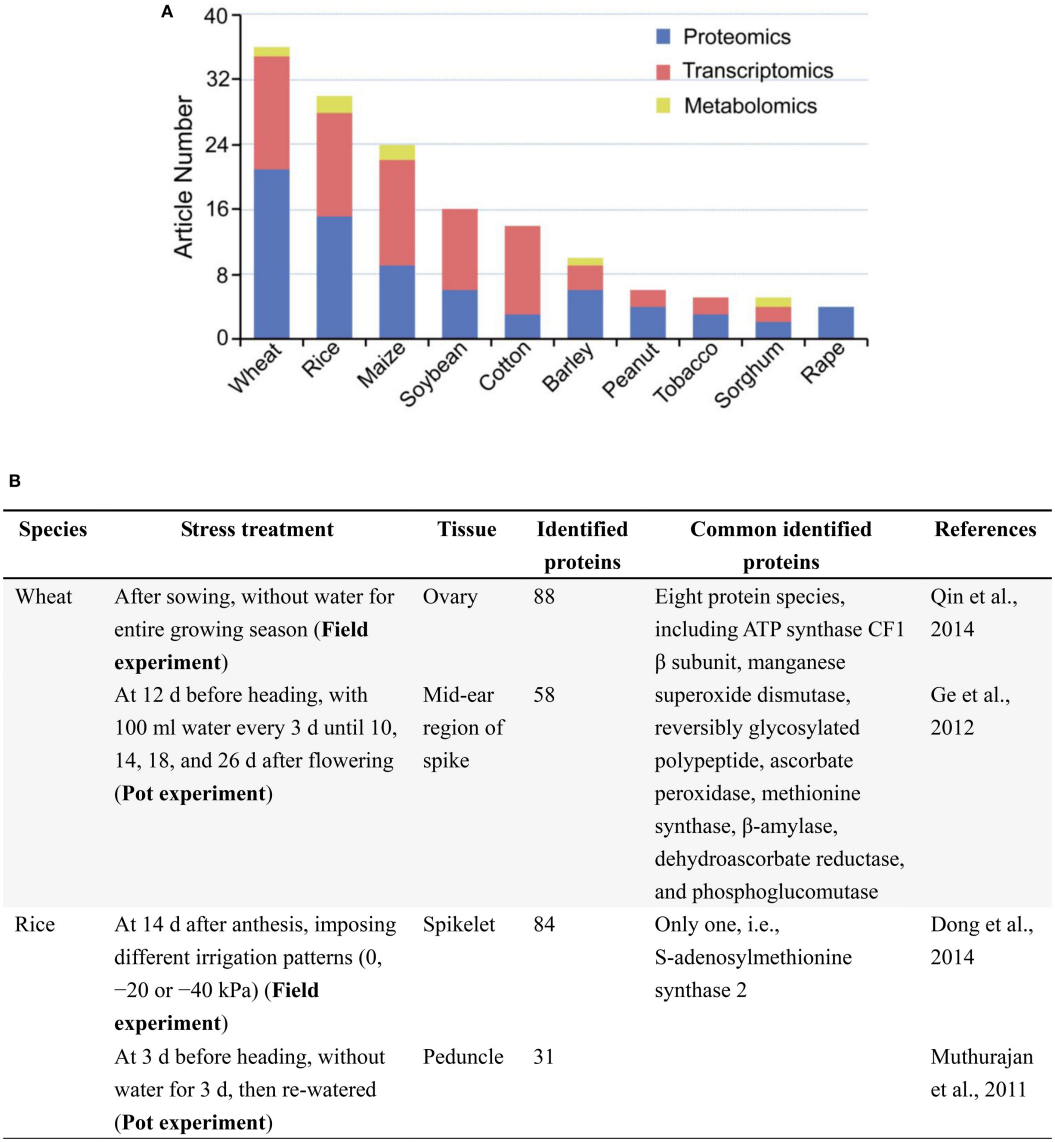
**A brief summary of omics analyses, especially proteomic analyses of drought responses in major crops. (A)** Original article numbers retrieved in the PubMed on proteomics, transcriptomics and metabolomics analyses. Search date: Oct 29, 2016. Keywords: drought, “crop name,” “omics method” (e.g., drought, wheat, proteomics/proteome). **(B)** Two examples of comparison between field and pot studies to find out common responsive proteins under drought. Common proteins share the same Gi number in NCBI database.

A search in PubMed (Oct 29, 2016) for original, omics articles on drought responses in major crops showed that proteomics, transcriptomics studies were much more than metabolomics studies (Figure 1A), and laboratory-based studies were overwhelming compared with those conducted in field (Supplementary Table 1). In all 151 original articles, only 11 reports on cotton, wheat, rice and maize were involved in field trials. Despite large amounts of biological information have been obtained from laboratory-grown seedlings, the results do not really reflect the performance of those in the field conditions, where the expression of drought tolerance trait of crops is most likely dependent on the interaction (abiotic and biotic) of genotype × environment. In other words, field assays are necessary to conclude results, and are closer to real conditions than the laboratory-studies. So, there is an urgent need to enhance omics analysis of crop responses to drought under the field, drought conditions. This is very valuable for crop improvement in the context of a changing climate and an increasing world population.

Since proteins are directly involved in plant stress responses, proteomic studies can eventually contribute to dissect the possible relationships between protein changes and plant stress tolerance. To date, numerous drought responsive-proteins have been identified with proteomic approaches in crops (Mousavi et al., 2016), which provide a wealth of data to elucidate the mechanisms of drought tolerance at the proteome level. However, proteomic analysis of crop plants under the drought field conditions is still scarce. Recently, we discussed the methodological defects in crop stress proteomics (Wu and Wang, 2016). Here, we take proteomic analysis as an example, aiming to critically analyze the experimental defects, and drought treatments in current crop proteomic analysis and propose the necessities of proteomic dissection of drought stress responses in crops under the field conditions. The discussion is expected to be applicable in transcriptomics and metabolomics research. While the focus is especially on drought stress, this discussion may also provide a reference to other abiotic stresses in crops.

## Drought treatments used in current crop proteomic research

In 74 original articles published over 2002–2016 in PubMed on proteomic analysis of drought responses in key crops, such as wheat, rice, maize, and soybean (Figure 1A), the stress treatments under the type of drought are generally classified into three groups: osmotic stress in laboratory or greenhouse, drought in controlled environments (e.g., pots in greenhouse), and drought in field conditions (Supplementary Table 1). Among 74 reports, only three are involved in field trials, mainly focused on organ/tissue (such as ovary and spikelet) of adult plants (Supplementary Table 1). Remarkably, a substantial portion of the studies are using osmotic stress mimicking drought stress usually with the leaves and/or roots of laboratory-grown seedlings as experimental materials and lack of real, drought stress in the field conditions (e.g., Hu et al., 2009, 2015), which may limit reproducibility and comparisons with other and/or future studies using field-grown crop plants. Though there are great differences (mainly in condition control) among field, pot, and laboratory studies, we compared and found out a limited number of common protein markers which are involved in the same metabolic pathways in response to drought stress (Figure 1B). Moreover, most stress treatments did not consider the mechanisms of drought responses (adaptation, damage, and recovery) in crops, which is a major concern about the experimental design in these proteomic analyses (Gilbert and Medina, 2016; Lyon et al., 2016). Because of the significant differences between osmotic stress and drought, using osmotic stress to mimic field drought is the widespread occurrence of methodological defect, thereby undermining the reproducibility and interpretation of the results from this fast-growing body of literature. Therefore, many studies do little to advance knowledge, except to add some species to the list of stress responsive proteins found previously, let alone their applications in the development of tailing crops for higher drought tolerance and yield potential.

One major cause of lack of field experiments is perhaps no fields available, which is a real, objective constraint on most research groups in the plant proteomics community. Hopefully, this problem can be solved through the cooperation between the scientists of plant proteomics with the research units involved in crop breeding. Another cause may be that adult plants are usually big, with long growth cycles, and difficult to culture in laboratories, whereas laboratory-grown seedlings are easily obtained for a “rapid” research driven by proteomic method toward the discovery of differentially abundant stress proteins.

Drought-prone environments are diverse, along with the biotic and abiotic stresses that affect crop yield under drought conditions. In general, only field trials allow for conclusive data on drought tolerance of target crops, as well as the value of any genes and the associated pathways for drought tolerance. However, field experiments need more replicates due to changeable environment conditions. Studies under controlled conditions (chamber, greenhouse) give valuable information that can be used to improve and reduce further field experiments (for instance reducing the number of genotypes/cultivars, or even doing a target search of proteins of interest). So, both laboratory experiments and field experiments should be complementary, therefore effort should be addressed in both ways. Besides, considering the importance of root traits, e.g., architecture and plasticity, for crop adaptation to water-deficit environments, proteomic analysis of field-grown roots will advance the knowledge of drought responses in crops.

## Enhancing experimental design of proteomic analysis of crops under the field, drought conditions

Proteomic dissection of drought responses in crops is usually initiated by detection of differentially abundant proteins after comparison between stressed and control plants. The relevant information on proteomics analysis has been established in the author guideline in Frontiers in Plant Science (Section: Plant Proteomics). In general, the proposed experimental designs for crop phenotyping (e.g., Campos et al., 2004; Cattivelli et al., 2008; Salekdeh et al., 2009) provide a good reference for proteomic analysis of drought responses in crops. Here, we only focus on several special requirements in proteomic analysis of crops grown under the field, drought conditions.

First of all, the experimental design should follow the mechanisms of drought adaptation in crops. Importantly, drought treatments should consider the level of drought intensity, speed of stress development, and duration specified by the hypothesized mechanism (Gilbert and Medina 2016). In some omic analyses, unfortunately, seedlings were just exposed to a series of “drought” stress with few or several physiological measures related to drought responses (Cattivelli et al., 2008), and then protein or mRNA abundance changes were measured, having no underlying biological reasons. In general, a field experiment should match the degree of manipulation of soil water deficit, stress, and damage to the nature of the mechanism being tested. For proteomic analysis, crops grown under controlled and field environments are subjected to reproducible drought treatment at specific developmental stages. A well-watered control is needed to monitor for evaluation of losses in growth and yield potential associated with drought tolerance. Comparison of performance in these contrasting environments provides the critical data required to drought tolerance in target crops.

Second, it is important to control stress level and timing for accurate evaluation of drought tolerance of target crops. Moreover, the detection of genotype × stress level interactions will provide essential evidence of the presence (and absence) of unique, adaptive mechanisms among genotypes. For the analysis of such interactions, relatively severe stress levels, more severe than those experienced in the target population of environments, are often imposed (Cattivelli et al., 2008). Besides, it should be considered that the different crop developmental stages show different sensitivity to drought. For example, maize is particularly susceptible to drought during its 2- to 3-week flowering period. A drought stress imposed at flowering may result in the widely variance of entries in time to silk, thus the most “tolerant” may simply be those that flower earlier than the mean.

Third, special attention should be paid to sampling in omics analysis. Compared to physiological and biochemical analyses, smaller amount of samples is needed for protein extraction and subsequent proteomics analysis, especially with sensitive detection approach. Thus, it is critical to take samples from representative plants in the community in the fields. The performance of the crop community under the field, drought conditions, plus physiological, and biochemical measurements of individual plants, can be used for the selection of representative plants. Depending on the source of target crops and research aim, the tissue samples from individual plants can be pooled or separated for use in omics analysis. For example, for phenotyping a crop mutant population to screen drought tolerant plants, samples from individual plants should be separately sampled and analyzed. Furthermore, the biological replicates should be at least three times to obtain proteomic data of confidence, which could never be replaced with technical replicates. Since proteomics is a statistics-based experimental science, biological replicates are vital for the enhanced confidence of proteomics data. In practice, random sampling of representative plants at different field spots can represent independent biological replicates.

Finally, experimental design should control within-replica variability. Particularly critical is the establishment of uniform stands to ensure evenness of drought level per plant. The level and timing of drought stress should be controlled in a manner relevant to target environment conditions (Campos et al., 2004; Cattivelli et al., 2008). To reduce the signal-to-noise ratio in field conditions, uniform plots with low spatial variability in soil properties are required. Additionally, the application of nutrients and the control of weeds/pests should be carried out precisely and uniformly. The use of rain shelters and supplementary irrigation can help to control the stress conditions and improve the quality of field experiments. Besides, owing to the variability in field environments, trial designs need to be conducted at multiple sites over multiple years to adequately replicate the results (Nuccio et al., 2015).

## Concluding remarks

Omics has proved to be a powerful technique for discovery of proteins and pathways that may be used to improve drought tolerance and productivity of crops in water-limited conditions. However, a wealth of biological information related to crop drought tolerance has not been translated from laboratory to field. Thus, scientists in plant omics community should make greater efforts to investigate drought responses of crops in field conditions, because such studies prove more valuable than laboratory-only studies. In addition, since molecular mechanisms underlying drought tolerant trait are complex and involve several levels of regulation, such as gene regulation, post-translational modifications, and protein interactions, the integration of omics approaches will enhance the quality and meaning of the derived biological information and get more valuable results for the development of drought-tolerant crops. The discussed concerns here may also apply to omics analysis of other abiotic stresses in crops.

## Author contributions

WW conceived the article. All authors contributed in manuscript writing, revising, and approved the final manuscript.

### Conflict of interest statement

The authors declare that the research was conducted in the absence of any commercial or financial relationships that could be construed as a potential conflict of interest.
