# Supplementary material for: Enhancing Omics Research of Crop Responses to Drought under Field Conditions
Source: Front Plant Sci. 2017 Feb 14;8:174. doi: 10.3389/fpls.2017.00174 (PMC5306382; doi:10.3389/fpls.2017.00174)
Supplement: Supplementary file 1 [file Table1.DOC]

**Supplementary Table 1 | A summary of drought stress treatments used in proteomics, transcriptomics and metabolomics analyses on major crops (search on Oct 29, 2016).**

| **Species** | **Stress treatment** | **Omics method** | | **Stage/Tissue** | **References** |
| --- | --- | --- | --- | --- | --- |
| Wheat | ***Field treatment*** | | | | |
|  | Withholding water for entire growing season | Proteomics | | Sowing, jointing, and flowering stages/Ovary | Qin et al., 2014 |
|  | ***Pots (in greenhouse or experimental plot) treatment*** | | | | |
|  | Withholding water for 48 h | | Proteomics | 1-week-old seedlings/Leaf | Cheng et al., 2016 |
|  | Withholding water with 30-35% soil water content (WC) | | Proteomics | Booting stage/Leaf, root | Qin et al., 2015 |
|  | Withholding water at 20% of field capacity (FC) | | Proteomics | Early-jointing stage/Leaf, root | Faghani et al., 2015 |
|  | Replenishing available soil water from 50% to 70% (Control plants to 100%) | | Proteomics | 73 d after sowing/Kernel | Giuliani et al., 2015 |
|  | Withholding water at 20% of FC | | Proteomics | Early-jointing stage/Leaf, root | Faghani et al., 2014 |
|  | Withholding water for 5 d until RWC to 20-25%, maintained for 3 d | | Proteomics | 15 d after anthesis/Leaf | Wang et al., 2014 |
|  | Withholding water at 1/3 of FC until physiological maturity | | Proteomics | At anthesis stage/Flag leaf | Peremarti et al., 2014 |
|  | Withholding water for 9 d | | Proteomics | 4-week-old seedlings/Leaf | Budak et al., 2013 |
|  | With 100 ml water every 3 d until 10, 14, 18, and 26 d after flowering | | Proteomics | 12 d before heading/Mid-ear region of spike | Ge et al., 2012 |
|  | With 1/3 SWC of the control group level until 10, 15, 20 and 25 d postanthesis | | Proteomics | After heading/Grain sample from middle spike | Jiang et al., 2012 |
|  | With 50% FC until physiological maturity | | Proteomics | 1/2 spikes extruded anthers/Stem | Bazargani et al., 2011 |
|  | Withholding water for 7 d and then for 6 d | | Proteomics | 3-leaf stage, anthesis stage/Kernel | Yang et al., 2011 |
|  | Withholding water until wilting, re-watered, then repeat once again | | Proteomics | 1-leaf stage/Leaf | Ford et al., 2011 |
|  | Irrigating at 150 ± 5 mm evaporation | | Proteomics | Booting stage/Seed | Hajheidari et al., 2007 |
|  | Placing seedlings on filter paper and exposing them to air for 0-24 h | | Transcriptomics | 10-d-old seedlings/Root | He et al., 2016 |
|  | Withholding water for 7 d | | Transcriptomics | 5−6-leaf stage/Root | Krugman et al., 2011 |
|  | Withholding water for 50% gravimetric WC | | Transcriptomics | Jointing stage/Leaf | Reddy et al., 2014 |
|  | Withholding water for 0, 2, 4 and 6 d | | Transcriptomics | 2-week-old seedlings/Leaf | Cheuk et al., 2016 |
|  | Withholding water for 6% SWC or 50% FC | | Transcriptomics | Booting stage/Leaf, head tissue | Liu et al., 2015 |
|  | Placing on dry filter paper, sampled every 30 min for different range of RWC | | Transcriptomics | 2-leaf stage/Leaf | Tavakol et al., 2014 |
|  | Withholding water for 8 d | | Transcriptomics | 10-week-old plants/Leaf | Krugman et al., 2010 |
|  | Withholding water for 9 d | | Transcriptomics | 4-week-old seedlings/Root | Akpinar et al., 2015 |
|  | Withholding water for 6 d | | Transcriptomics | Anthesis stage/Leaf | Habash et al., 2014 |
|  | Withholding water for 18% and 12.5% of SWC | | Transcriptomics | After anthesis/Leaf | Aprile et al., 2009 |
|  | Leaving on paper towels for 4 to 8 h | | Transcriptomics | 6-week-old seedlings/Root, leaf | Ergen et al., 2009 |
|  | Dehydrating on dry paper for 4 d | | Transcriptomics | 7-d-old seedlings/Leaf | Kurahashi et al., 2009 |
|  | Withholding water for 7 d | | Metabolomics | 5−6-leaf stage/Root | Krugman et al., 2011 |
|  | ***Laboratory osmotic treatment*** | | | | |
|  | 0, 15%, 20%, 25%, and 30% PEG 6000 for 48 h | Proteomics | | 3-leaf stage/Leaf | Cheng et al., 2015 |
|  | 20% PEG 6000 for 48 h | Proteomics | | 3-leaf stage/Root, leaf | Liu et al., 2015 |
|  | 20% PEG 6000 (−0.75 MPa) for 48 h, then recovered for 48 h | Proteomics | | 3-leaf stage/Root, leaf, etc. | Hao et al., 2015 |
|  | 20% PEG 6000 for 48 h | Proteomics | | 3-leaf stage/Leaf | Zhang et al., 2014 |
|  | 100 μM ABA for 6 h | Proteomics | | 10-d-old seedlings/Root | Alvarez et al., 2014 |
|  | 18% PEG 6000 for 24 h | Proteomics | | 2-leaf stage/Leaf, root | Peng et al., 2009 |
|  | PEG 6000 for 3, 12, and 24 h | Transcriptomics | | 10-d-old seedlings/Leaf | Baloglu et al., 2014 |
|  | 20% PEG 6000 for 1 and 6 h | Transcriptomics | | 1-week-old seedlings/Leaf | Liu et al., 2015 |
| Rice | ***Field treatment*** | | | | |
|  | Imposing different irrigation patterns (0, −20 or −40 kPa) | Proteomics | | 14 d after anthesis/Spikelet | Dong et al., 2014 |
|  | Stopping irrigation at 35 d after sowing | Metabolomics | | Flowering stage/Spikelet, leaf, stem, root | Raorane et al., 2015 |
|  | ***Pots (in greenhouse or experimental plot)*** | | | | |
|  | Withholding water for 7 d | Proteomics | | 4-week-old seedlings/Leaf | Wu et al., 2016 |
|  | Withholding water for 7 d | Proteomics | | 3 d before heading/Root | Paul et al., 2015 |
|  | With 50% reposition of the water lost daily | Proteomics | | Anthesis stage/Leaf | Rabello et al., 2014 |
|  | Withholding water for 14 d | Proteomics | | 27-d-old seedlings/Shoot | Mirzaei et al., 2014 |
|  | Withholding water for 14 d | Proteomics | | 35-d-old seedlings/Leaf | Mirzaei et al., 2012a |
|  | Withholding water for 14 d | Proteomics | | 27-d-old seedlings/Root | Mirzaei et al., 2012b |
|  | Withholding water for 20 d | Proteomics | | Booting stage/Leaf | Ji et al., 2012 |
|  | Withholding water for a period of 1-9 d | Proteomics | | 30 d after sowing/Leaf | Shu et al., 2011 |
|  | Removing standing water and withholding water for 3 d, then re-watered | Proteomics | | 3 d before heading/Peduncle | Muthurajan et al., 2011 |
|  | Withholding water for 5 d, and thereafter restored until heading | Proteomics | | 3 d before heading/Anther | Liu and Bennett, 2011 |
|  | Stopping water for 4 d | Proteomics | | 2-week-old seedlings/Leaf | Ke et al., 2009 |
|  | With 50% reposition of the water lost daily | Proteomics | | Anthesis stage/Root | Rabello et al., 2008 |
|  | Withholding water from 2 to 6 d | Proteomics | | 2-week-old seedlings/Leaf sheath | Ali and Komatsu, 2006 |
|  | Over 23 d of transpiration without watering | Proteomics | | 3-week-old seedlings/Leaf | Salekdeh et al., 2002 |
|  | Withholding water supply till visible leaf rolling appeared | Transcriptomics | | 14-d-old seedlings/Leaf | Lenka et al., 2011 |
|  | Withholding water for 0.2 and 0.5 fraction of transpirable soil water (FTSW) | Transcriptomics | | Reproductive stage/Leaf | Moumeni et al., 2015 |
|  | Withholding water until leaf RWC to 65%-75% | Transcriptomics | | Tillering, panicle elongation, booting stage/Leaf, root | Wang et al., 2011 |
|  | Withholding water for 0.5 FTSW | Transcriptomics | | 10 d before heading/Leaf | Ereful et al., 2016 |
|  | Withholding water for 1, 2, and 3 d | Transcriptomics | | 10-leaf stage/Leaf | Chung et al., 2016 |
|  | 25% SWC for one week | Transcriptomics | | At stage In5/Floret | Jin et al., 2013 |
|  | Dry until target FTSW and then re-watered daily to maintain target FTSW | Transcriptomics | | V6/V7 stage/Leaf | Cal et al., 2013 |
|  | Withholding water for one week | Transcriptomics | | 4-week-old seedlings/Leaf | Zhang et al., 2016 |
|  | Withholding water for 4 d | Transcriptomics | | 26 d after sowing/Leaf | Degenkolbe et al., 2013 |
|  | Withholding water to 0.2 and 0.5 FTSW | Transcriptomics | | 35 d after seeding/Root | Moumeni et al., 2011 |
|  | Withholding water for two weeks, then re-watered for one week | Metabolomics | | Tillers first appeared/Kernel | Nam et al., 2016 |
|  | ***Laboratory osmotic treatment*** | | | | |
|  | 15% PEG 6000 for 24 h | Transcriptomics | | 3-week-old seedlings/Root, shoot | Tian et al., 2015 |
|  | 25 % PEG for 1 h | Transcriptomics | | 21-d-old seedlings/Leaf | Lima et al., 2015 |
|  | 15% PEG 6000 | Transcriptomics | | 3-leaf stage/Leaf | Zhang et al., 2015 |
| Maize | ***Field treatment*** | | | | |
|  | Withholding water to 50% of applied irrigation in the well-watered control until 35 d after pollination | Proteomics | | 14 d after pollination/Kernel | Yang et al., 2014 |
|  | Withholding water for 11, 18, 27, and 32 d | Transcriptomics | | After stage V8/Ear, tassel, leaf | Thatcher et al., 2016 |
|  | With soil moisture content (SMC) about 16% | Transcriptomics | | 3 weeks before flowering/Kernel | Marino et al., 2009 |
|  | Stopping irrigation | Metabolomics | | Flowering stage/Leaf | Obata et al., 2015 |
|  | ***Pots (in greenhouse or experimental plot)*** | | | | |
|  | Withholding water for 6 d | Proteomics | | 11-leaf-stage/Leaf | Dworak et al., 2016 |
|  | Withholding water for 6 d | Proteomics | | 4-leaf stage/Leaf | Benešová et al., 2012 |
|  | Withholding water for 7, 10 and 12 d | Proteomics | | 15 d after sowing/Root (Xylem sap) | Alvarez et al., 2008 |
|  | Withholding water for different periods of time | Proteomics | | 5-leaf stage/Leaf | Vincent et al., 2005 |
|  | Without water until indications of withering, then re-watered | Transcriptomics | | 3-leaf stage/Leaf | Min et al., 2016 |
|  | With 25% of disposable water at 0.1dS/m salt concentration for 10 d | Transcriptomics | | V5/V6 stage/Leaf | Forestan et al., 2016 |
|  | Withholding water for 5 d | Transcriptomics | | 5-week-old seedlings/Leaf, stem, root | Casaretto et al., 2016 |
|  | With 25% of disposable water at 0.1 dS/m salt concentration for 10 d | Transcriptomics | | V5/V6 stage/Leaf, shoot | Lunardon et al., 2016 |
|  | With 35% of relative SWC for one week, then kept stable for 2-3 d | Transcriptomics | | 3-week-old seedlings/Leaf, stem, root | Liu et al., 2015 |
|  | Adjusting water supply for 1 and 7 d | Transcriptomics | | Silking stage/Leaf | Yue et al., 2008 |
|  | Withholding water for 3 or 4 d | Transcriptomics | | Reproductive stage/Leaf, ovary | Kakumanu et al., 2012 |
|  | Withholding water for 10 d | Transcriptomics | | 2-week-old seedlings/Leaf | Lei et al., 2015 |
|  | Withholding water to different levels of seedling leaf RWC, then re-watered | Transcriptomics | | 3-leaf seedlings/Shoot | Zheng et al., 2010 |
|  | Withholding nutrient solution | Metabolomics | | 17 d after sowing/Leaf | Sicher et al., 2012 |
|  | ***Laboratory osmotic treatment*** | | | | |
|  | PEG solution (−0.7 MPa) for 8 h | Proteomics | | 5-leaf stage/Leaf | Hu et al., 2015a |
|  | PEG solution (−0.7 MPa) for 8 h | Proteomics | | 2-week-old seedlings/Leaf | Hu et al., 2015b |
|  | Mannitol (−1.0 MPa) solution for 8 h | Proteomics | | 2-week-old seedlings/Leaf | Hu et al., 2012 |
|  | 16% PEG 6000 solution (−0.5 MPa) for 17, 24 and 48 h | Proteomics | | 7-leaf stage/Leaf | Hu et al., 2009 |
|  | PEG 8000 for 6 h | Transcriptomics | | 4-d-old seedlings/Root | Opitz et al., 2016 |
|  | PEG 8000 (-0.2 MPa and -0.8 MPa) for 6 and 24 h | Transcriptomics | | 4- to 5-d-old seedlings/Root | Opitz et al., 2014 |
| Soybean | ***Pots (in greenhouse or experimental plot)*** | | | | |
|  | Withholding water for 4 d, then re-watered for 4 d | Proteomics | | 2-d-old seedlings/Root (tips), hypocotyl | Khan and Komatsu, 2016 |
|  | Withholding water for 2 d | Proteomics | | 2-d-old seedlings/Root tip | Wang and Komatsu, 2016 |
|  | Withholding water for 7 d | Proteomics | | The second trifoliate leaves emerged/Leaf | Das et al., 2016 |
|  | Withholding water for 1 or 2 d | Proteomics | | 2-4-d-old seedlings/Root tip | Wang et al., 2016 |
|  | Withholding water for 2 d | Proteomics | | 2-d-old seedlings/Root | Oh and Komatsu, 2015 |
|  | Withholding water for 4 d | Proteomics | | 3-d-old seedlings/Leaf, root, hypocotyl | Mohammadi et al., 2012 |
|  | Withdrawing water for 15 d | Transcriptomics | | V5 stage/Leaf | Prince et al., 2015 |
|  | Without irrigation till 30% of soil field capacity | Transcriptomics | | 14-d-old plant/Leaf | Rodrigues et al., 2015 |
|  | Withholding water for 5 d and 19 d, then re-watered for 2 d | Transcriptomics | | V3 stage/Root | Song et al., 2016 |
|  | Exposing whole plants to air for 0, 6, 12, and 24 h | Transcriptomics | | R2 stage of flowering/Leaf | Shin et al., 2015 |
|  | Withdrawing water for 7 d | Transcriptomics | | V4 stage/Leaf | Chen et al., 2016 |
|  | Withdrawing water for 7 d | Transcriptomics | | V4 stage/Root | Guimarães-Dias et al., 2012 |
|  | Exposing seedlings to air for 25, 50, 75, 100, 125 and 150 min | Transcriptomics | | V4 stage/Root | Rodrigues et al., 2012 |
|  | Transferring seedlings to empty boxes for 30 min, 1 h, 2 h, 3 h and 5 h | Transcriptomics | | 30-d-old plant/Leaf, root | Tripathi et al., 2015 |
|  | Withholding water till SMC at 40–45% | Transcriptomics | | V6 stage/Leaf | Le et al., 2012 |
|  | ***Laboratory osmotic treatment*** | | | | |
|  | 2% PEG for 48 h | Transcriptomics | | 4-leaf stage/Root | Li et al., 2011 |
| Barely | ***Pots (in greenhouse or experimental plot)*** | | | | |
|  | Withholding water at 3.2 pF (158.5 kPa) of SMC for 10 d | Proteomics | | 3-leaf stage/Leaf, root | Chmielewska et al., 2016 |
|  | Withholding water at 65, 35 and 30% of soil water capacity for 10 d | Proteomics | | 2-leaf stage/Crown | Vítámvás et al., 2015 |
|  | Withholding water at 10% and 4% of SMC for 20 d, then recovered | Proteomics | | 2-leaf stage/Leaf | Wang et al., 2015 |
|  | Withholding water at 15% of FC until plant physiological maturity | Proteomics | | At heading stage/Leaf | Rollins et al., 2013 |
|  | Withholding water for 3 d | Proteomics | | 3-d-old seedlings/Shoot | Kausar et al., 2013 |
|  | Withholding water for 7 d | Proteomics | | 7-d-old seedlings/Leaf, root | Wendelboe and Morris, 2012 |
|  | Without watering for 4 d | Transcriptomics | | 4-week-old seedlings/Leaf, root | Vojta et al., 2016 |
|  | Without irrigation till 33.4%, 27.5%, 21.1%, 15.5%, 9.8% and 4.8% of relative SMC for 8 d | Transcriptomics | | 18 d after sowing/Leaf | Zeng et al., 2016 |
|  | With 1.5% and 3% volumetric WC for 10 d and 4 d, respectively | Transcriptomics | | 11 d after sowing/Leaf, root | Kwasniewski et al., 2016 |
|  | With SMC to 3.2 pF (158.5 kPa) | Metabolomics | | 3-leaf stage/Leaf, root | Chmielewska et al., 2016 |
| Peanut | ***Pots (in greenhouse or experimental plot)*** | | | | |
|  | Withholding water for 15 d | Proteomics | | 50-d-old plants/Leaf | Katam et al., 2016 |
|  | Withholding water for 5 or 6 d | Proteomics | | 30 d after seed emergence/Leaf | Akkasaeng et al., 2015 |
|  | Withholding water until seed maturity | Proteomics | | 57 d after sowing/Kernel | Senakoon et al., 2015 |
|  | With sprinkler irrigation regime for 25 mm of water per week until 110 d after sowing | Proteomics | | 14-d-old seedlings/Pod | Kottapalli et al., 2013 |
|  | With different normalized tanspiration ratio (0.76, 0.73, 0.57, 0.43 and 0.40) | Transcriptomics | | 3-month-old plants/Leaf, root | Guimarães et al., 2012 |
|  | With different normalized transpiration rate values | Transcriptomics | | 3-month-old plants/Leaf, root | Brasileiro et al., 2015 |
| Rape | ***Pots (in greenhouse or experimental plot)*** | | | | |
|  | Removing plants from pot together with soil, then air-dried for 24 and 48 h | Proteomics | | 3-week-old plants/Whole plant except root | Kwon et al., 2016 |
|  | No watering for 3, 5, 7, 10, 12, or 14 d | Proteomics | | 6-week-old plants/Leaf | Koh et al., 2015 |
|  | Withholding water for 7 d | Proteomics | | 7-d-old seedlings/Root | Mohammadi et al., 2012 |
|  | ***Laboratory osmotic treatment*** | | | | |
|  | 25% PEG 6000 solution (−1.0 MPa) for 4 h | Proteomics | | 15-d-old seedlings/Leaf | Luo et al., 2015 |
| Tobacco | ***Pots (in greenhouse or experimental plot)*** | | | | |
|  | Stopping watering for 2 d | Proteomics | | 40-d-old plants/Leaf | Xie et al., 2016 |
|  | Withholding water for 12 d | Proteomics | | 6–8-week-old plants/Leaf | Gharechahi et al., 2015 |
|  | Dehydrating for 20, 40, 60, 120 and 240 min | Transcriptomics | | 2-week-old plants/Leaf, root | Rabara et al., 2015a |
|  | Dehydrating for 20, 40, 60, 120 and 240 min | Transcriptomics | | 2-week-old plants/Leaf, root | Rabara et al., 2015b |
|  | ***Laboratory osmotic treatment*** | | | | |
|  | 100, 150, and 200 mM mannitol for 72 h | Proteomics | | 2-week-old seedlings/Leaf | Kumar et al., 2014 |
| Cotton | ***Field treatment*** | | | | |
|  | Withholding water for 7 d | Transcriptomics | | 2-week-old seedlings/Leaf | Ranjan et al., 2012 |
|  | Without irrigation for 4 weeks | Transcriptomics | | Reproductive growth stage/Leaf, root | Park et al., 2012 |
|  | Withholding water for 18-19 d | Transcriptomics | | 70 d after sowing/Leaf | Padmalatha et al., 2012 |
|  | Without irrigation till leaf water potential to -0.2 MPa or greater for 4 weeks | Transcriptomics | | Reproductive growth stage/Root | Bowman et al., 2013 |
|  | ***Pots (in greenhouse or experimental plot)*** | | | | |
|  | Withholding water for 10 d, then re-watered for 5 d | Proteomics | | 6–8 sympodial branches were flowering/Fiber | Zheng et al., 2014 |
|  | Withholding water at 75, 50 and 35% RWC for 7 d | Proteomics | | 3-week-old plant/Leaf | Deeba et al., 2012 |
|  | Withholding water for 7 d | Transcriptomics | | 4-week-old plants/Root | Ranjan et al., 2012 |
|  | Withholding water for 15 d | Transcriptomics | | One month old plants/Leaf | Singh et al., 2015 |
|  | Withholding water for 15 d, then recovered for 5 d | Transcriptomics | | One month old plants/Root | Singh et al., 2016 |
|  | Without irrigation until leaf water potentials averaged –8.7 bars (±0.37 SE) | Transcriptomics | | 12-w-old plants/Leaf, root | Payton et al., 2011 |
|  | Placing on dry filter paper for 2 d | Transcriptomics | | 6–8-d-old plants/Leaf, root | Dong et al., 2011 |
|  | Withholding water till the RWC dropped to about 7% | Transcriptomics | | Trefoil stage/Leaf | Lu et al., 2016 |
|  | ***Laboratory osmotic treatment*** | | | | |
|  | 15% PEG 6000 solution for 24 h | Proteomics | | 3-leaf stage/Root | Zhang et al., 2016 |
|  | 200 mM mannitol for 10 d | Transcriptomics | | 14-d-old plants/Seedling | Zhu et al., 2013 |
| Sorghum | ***Pots (in greenhouse or experimental plot)*** | | | | |
|  | Leaving soil to the level of 10% FC and further withholding water for 12 d | Proteomics | | 5-leaf stage/Leaf | Jedmowski et al., 2014 |
|  | No water supply for 96 h | Proteomics | | 7-d-old plants/Leaf | Sharma et al., 2012 |
|  | Withholding water till soil water potential reached to 0.13, 0.44, and 1.38 MPa | Transcriptomics | | 4-leaf stage/Leaf | Pasini et al., 2014 |
|  | Withholding irrigation until the leaf RWC reached to about 60-65% | Transcriptomics | | One month old plants/Leaf | Katiyar et al., 2015 |
|  | Reducing 75% water supply | Metabolomics | | 60-d-old plants/Leaf, culm, root, grain | De Souza et al., 2015 |

**References**

Akkasaeng, C., Tantisuwichwong, N., Ngamhui, N. O., Roytrakul, S., Jogloy, S., and Pathanothai, A. (2015). Changes in protein expression in peanut leaves in the response to progressive water stress. *Pak. J. Biol. Sci*. 18, 19-26

Akpinar, B. A., Kantar, M., and Budak, H. (2015). Root precursors of microRNAs in wild emmer and modern wheats show major differences in response to drought stress. *Funct. Integr. Genomics*. 15, 587-598. doi: 10.1007/s10142-015-0453-0

Ali, G. M., and Komatsu, S. (2006). Proteomic analysis of rice leaf sheath during drought stress. *J. Proteome Res*. 5, 396-403

Alvarez, S., Roy Choudhury, S., and Pandey, S. (2014). Comparative quantitative proteomics analysis of the ABA response of roots of drought-sensitive and drought-tolerant wheat varieties identifies proteomic signatures of drought adaptability. *J. Proteome Res*. 13, 1688-1701. doi: 10.1021/pr401165b

Alvarez, S., Marsh, E. L., Schroeder, S. G., and Schachtman, D. P. (2008). Metabolomic and proteomic changes in the xylem sap of maize under drought. *Plant Cell Environ*. 31, 325-340

Aprile, A., Mastrangelo, A. M., De Leonardis, A. M., Galiba, G., Roncaglia, E., Ferrari, F., et al. (2009). Transcriptional profiling in response to terminal drought stress reveals differential responses along the wheat genome. *BMC Genomics*. 10, 279. doi: 10.1186/1471-2164-10-279

Baloglu, M. C., Inal, B., Kavas, M., and Unver, T. (2014). Diverse expression pattern of wheat transcription factors against abiotic stresses in wheat species. *Gene*. 550, 117-122. doi: 10.1016/j.gene.2014.08.025

Bazargani, M. M., Sarhadi, E., Bushehri, A. A., Matros, A., Mock, H. P., Naghavi, M. R., et al. (2011). A proteomics view on the role of drought-induced senescence and oxidative stress defense in enhanced stem reserves remobilization in wheat. *J. Proteomics*. 74, 1959-1973. doi: 10.1016/j.jprot.2011.05.015

Benešová, M., Holá, D., Fischer, L., Jedelský, P. L., Hnilička, F., Wilhelmová, N., et al. (2012). The physiology and proteomics of drought tolerance in maize: Early stomatal closure as a cause of lower tolerance to short-term dehydration? *PLoS One* 7, e38017. doi: 10.1371/journal.pone.0038017

Bowman, M. J., Park, W., Bauer, P. J., Udall, J. A., Page, J. T., Raney, J., et al. (2013). RNA-Seq transcriptome profiling of upland cotton (Gossypium hirsutum L.) root tissue under water-deficit stress. *PLoS One* 8, e82634. doi: 10.1371/journal.pone.0082634

Brasileiro, A. C., Morgante, C. V., Araujo, A. C., Leal-Bertioli, S. C., Silva, A. K., Martins, A. C., et al. (2015). Transcriptome profiling of wild Arachis from water-limited environments uncovers drought tolerance candidate genes. *Plant Mol. Biol. Report.* 33, 1876-1892. doi: 10.1007/s11105-015-0882-x

Budak, H., Akpinar, B. A., Unver, T., and Turktas, M. (2013). Proteome changes in wild and modern wheat leaves upon drought stress by two-dimensional electrophoresis and nanoLC-ESI-MS/MS. *Plant Mol. Biol*. 83, 89-103. doi: 10.1007/s11103-013-0024-5

Cal, A. J., Liu, D., Mauleon, R., Hsing, Y. I., and Serraj, R. (2013). Transcriptome profiling of leaf elongation zone under drought in contrasting rice cultivars. *PLoS One* 8, e54537. doi: 10.1371/journal.pone.0054537

Casaretto, J. A., El-Kereamy, A., Zeng, B., Stiegelmeyer, S. M., Chen, X., Bi, Y. M., et al. (2016). Expression of OsMYB55 in maize activates stress-responsive genes and enhances heat and drought tolerance. *BMC Genomics* 17, 312. doi: 10.1186/s12864-016-2659-5

Chen, W., Yao, Q., Patil, G. B., Agarwal, G., Deshmukh, R. K., Lin, L., et al. (2016). Identification and comparative analysis of differential gene expression in soybean leaf tissue under drought and flooding stress revealed by RNA-Seq. *Front. Plant Sci.* 7, 1044. doi: 10.3389/fpls.2016.01044

Cheng, L., Wang, Y., He, Q., Li, H., Zhang, X., and Zhang, F. (2016). Comparative proteomics illustrates the complexity of drought resistance mechanisms in two wheat (*Triticum aestivum* L.) cultivars under dehydration and rehydration. *BMC Plant Biol*. 16, 188. doi: 10.1186/s12870-016-0871-8

Cheng, Z., Dong, K., Ge, P., Bian, Y., Dong, L., Deng, X., et al. (2015). Identification of leaf proteins differentially accumulated between wheat cultivars distinct in their levels of drought tolerance. *PLoS One* 10, e0125302. doi: 10.1371/journal.pone.0125302

Cheuk, A., and Houde, M. (2016). Genome wide identification of C1-2i zinc finger proteins and their response to abiotic stress in hexaploid wheat. *Mol. Genet. Genomics* 291, 873-890. doi: 10.1007/s00438-015-1152-1

Chmielewska, K., Rodziewicz, P., Swarcewicz, B., Sawikowska, A., Krajewski, P., Marczak, Ł., et al. (2016). Analysis of drought-induced proteomic and metabolomic changes in barley (*Hordeum vulgare* L.) leaves and roots unravels some aspects of biochemical mechanisms involved in drought tolerance. *Front. Plant Sci.* 7, 1108. doi: 10.3389/fpls.2016.01108

Chung, P. J., Jung, H., Jeong, D. H., Ha, S. H., Choi, Y. D., and Kim, J. K. (2016). Transcriptome profiling of drought responsive noncoding RNAs and their target genes in rice. *BMC Genomics* 17, 563. doi: 10.1186/s12864-016-2997-3

Das, A., Eldakak, M., Paudel, B., Kim, D. W., Hemmati, H., Basu, C., et al. (2016). Leaf proteome analysis reveals prospective drought and heat stress response mechanisms in soybean. *Biomed Res. Int*. 2016, 6021047. doi: 10.1155/2016/6021047

De Souza, A. P., Cocuron, J. C., Garcia, A. C., Alonso, A. P., and Buckeridge, M. S. (2015). Changes in whole-plant metabolism during the grain-filling stage in sorghum grown under elevated CO_2_ and drought. *Plant Physiol.*169, 1755-65. doi: 10.1104/pp.15.01054

Deeba, F., Pandey, A. K., Ranjan, S., Mishra, A., Singh, R., Sharma, Y. K., et al. (2012). Physiological and proteomic responses of cotton (*Gossypium herbaceum* L.) to drought stress. *Plant Physiol. Biochem*. 53, 6-18. doi: 10.1016/j.plaphy.2012.01.002

Degenkolbe, T., Do, P. T., Kopka, J., Zuther, E., Hincha, D. K., and Köhl, K. I. (2013). Identification of drought tolerance markers in a diverse population of rice cultivars by expression and metabolite profiling. *PLoS One* 8, e63637. doi: 10.1371/journal.pone.0063637

Dong, M., Gu, J., Zhang, L., Chen, P., Liu, T., Deng, J., et al. (2014). Comparative proteomics analysis of superior and inferior spikelets in hybrid rice during grain filling and response of inferior spikelets to drought stress using isobaric tags for relative and absolute quantification. *J. Proteomics* 109, 382-399. doi: 10.1016/j.jprot.2014.07.001

Dong, S., and [Adams, K. L](https://www.ncbi.nlm.nih.gov/pubmed/?term=Adams KL%5BAuthor%5D&cauthor=true&cauthor_uid=21361962). (2011). Differential contributions to the transcriptome of duplicated genes in response to abiotic stresses in natural and synthetic polyploids. [*New Phytol.*](https://www.ncbi.nlm.nih.gov/pubmed/?term=Differential+contributions+to+the+transcriptome+of+duplicated+genes+in+response+to+abiotic+stresses+in+natural+and+synthetic+polyploids)190, 1045-1057. doi: 10.1111/j.1469-8137.2011.03650.x

Dworak, A., Nykiel, M., Walczak, B., Miazek, A., Szworst-Łupina, D., Zagdańska, B., et al. (2016). Maize proteomic responses to separate or overlapping soil drought and two-spotted spider mite stresses. *Planta* 244, 939-960. doi: 10.1007/s00425-016-2559-6

Ereful, N. C., Liu, L. Y., Tsai, E., Kao, S. M., Dixit, S., Mauleon, R., et al. (2016). Analysis of allelic imbalance in rice hybrids under water stress and association of asymmetrically expressed genes with drought-response QTLs. *Rice (N Y)*. 9, 50. doi: 10.1186/s12284-016-0123-4

Ergen, N. Z., Thimmapuram, J., Bohnert, H. J., and Budak, H. (2009). Transcriptome pathways unique to dehydration tolerant relatives of modern wheat. *Funct. Integr. Genomic*. 9, 377-396. doi: 10.1007/s10142-009-0123-1

Faghani, E., Gharechahi, J., Komatsu, S., Mirzaei, M., Khavarinejad, R. A., Najafi, F., et al. (2014). Data in support of comparative physiology and proteomic analysis of two wheat genotypes contrasting in drought tolerance. *Data Brief* 2, 26-28. doi: 10.1016/j.dib.2014.11.001

Faghani, E., Gharechahi, J., Komatsu, S., Mirzaei, M., Khavarinejad, R. A., Najafi, F., et al. (2015). Comparative physiology and proteomic analysis of two wheat genotypes contrasting in drought tolerance. *J. Proteomics* 114, 1-15. doi: 10.1016/j.jprot.2014.10.018

Ford, K., L., Cassin, A., and Bacic, A. (2011). Quantitative proteomic analysis of wheat cultivars with differing drought stress tolerance. *Front. Plant Sci*. 2, 44. doi: 10.3389/fpls.2011.00044

Forestan, C., Aiese Cigliano, R., Farinati, S., Lunardon, A., Sanseverino, W., and Varotto, S. (2016). Stress-induced and epigenetic-mediated maize transcriptome regulation study by means of transcriptome reannotation and differential expression analysis. *Sci. Rep*. 6, 30446. doi: 10.1038/srep30446

Ge, P., Ma, C., Wang, S., Gao, L., Li, X., Guo, G., et al. (2012). Comparative proteomic analysis of grain development in two spring wheat varieties under drought stress. *Anal. Bioanal. Chem*. 402, 1297-1313. doi: 10.1007/s00216-011-5532-z

Gharechahi, J., Hajirezaei, M. R., and Salekdeh, G. H. (2015). Comparative proteomic analysis of tobacco expressing cyanobacterial flavodoxin and its wild type under drought stress. *J. Plant Physiol*. 175, 48-58. doi: 10.1016/j.jplph.2014.11.001

Giuliani, M. M., Palermo, C., De Santis, M. A., Mentana, A., Pompa, M., Giuzio, L., et al. (2015). Differential expression of durum wheat gluten proteome under water stress during grain filling. *J. Agric. Food Chem*. 63, 6501-6512. doi: 10.1021/acs .jafc.5b01635

Guimarães-Dias, F., Neves-Borges, A. C., Viana, A. A., Mesquita, R. O., Romano, E., de FátimaGrossi-de-Sá, M., et al. (2012). Expression analysis in response to drought stress in soybean: Shedding light on the regulation of metabolic pathway genes. *Genet. Mol. Biol.* 35, 222-232. doi: 10.1590/S1415-47572012000200004

Guimarães, P. M., Brasileiro, A. C., Morgante, C. V., Martins, A. C., Pappas, G., Silva, O. B. Jr., et al. (2012). Global transcriptome analysis of two wild relatives of peanut under drought and fungi infection. *BMC Genomics* 13, 387. doi: 10.1186/1471-2164-13-387

Habash, D. Z., Baudo, M., Hindle, M., Powers, S. J., Defoin-Platel, M., Mitchell, R., et al. (2014). Systems responses to progressive water stress in durum wheat. *PLoS One*. 9, e108431. doi: 10.1371/journal.pone.0108431

Hao, P., Zhu, J., Gu, A., Lv, D., Ge, P., Chen, G., et al. (2015). An integrative proteome analysis of different seedling organs in tolerant and sensitive wheat cultivars under drought stress and recovery. *Proteomics* 15, 1544-1563. doi: 10.1002/pmic.201400179

He, G. H., Xu, J. Y., Wang, Y. X., Liu, J. M., Li, P. S., Chen, M., et al. (2016). Drought-responsive WRKY transcription factor genes *TaWRKY1* and *TaWRKY33* from wheat confer drought and/or heat resistance in Arabidopsis. *BMC Plant Biol.* 16, 116. doi: 10.1186/s12870-016-0806-4

Hajheidari, M., Eivazi, A., Buchanan, B. B., Wong, J. H., Majidi, I., and Salekdeh, G. H. (2007). Proteomics uncovers a role for redox in drought tolerance in wheat. *J. Proteome Res.* 6, 1451-1460

Hu, X., Wu, L., Zhao, F., Zhang, D., Li, N., Zhu, G., et al. (2015a). Phosphoproteomic analysis of the response of maize leaves to drought, heat and their combination stress. *Front. Plant Sci.* 6, 298. doi: 10.3389/fpls.2015.00298

Hu, X., Li, N., Wu, L., Li, C., Li, C., Zhang, L., et al. (2015b). Quantitative iTRAQ-based proteomic analysis of phosphoproteins and ABA-regulated phosphoproteins in maize leaves under osmotic stress. *Sci. Rep*. 5, 15626. doi: 10.1038/srep15626

Hu, X., Wu, X., Li, C., Lu, M., Liu, T., Wang, Y., et al. (2012). Abscisic acid refines the synthesis of chloroplast proteins in maize (*Zea mays*) in response to drought and light. *PLoS One* 7, e49500. doi: 10.1371/journal.pone.0049500

Hu, Y., Li, W. C., Xu, Y. Q., Li, G. J., Liao, Y., and Fu, F. L. (2009). Differential expression of candidate genes for lignin biosynthesis under drought stress in maize leaves. *J. Appl. Genet*. 50, 213-223. doi: 10.1007/BF03195675

Jedmowski, C., Ashoub, A., Beckhaus, T., Berberich, T., Karas, M., and Brüggemann, W. (2014). Comparative analysis of sorghum bicolor proteome in response to drought stress and following recovery. *Int. J. Proteomics* 2014, 395905. doi: 10.1155/2014/395905

Ji, K., Wang, Y., Sun, W., Lou, Q., Mei, H., Shen, S., et al. (2012). Drought-responsive mechanisms in rice genotypes with contrasting drought tolerance during reproductive stage. *J. Plant Physiol*. 169, 336-344. doi: 10.1016/j.jplph.2011.10.010

Jiang, S. S., Liang, X. N., Li, X., Wang, S. L., Lv, D. W., Ma, C. Y., et al. (2012). Wheat drought-responsive grain proteome analysis by linear and nonlinear 2-DE and MALDI-TOF mass spectrometry. *Int. J. Mol. Sci*. 13, 16065-16083. doi: 10.3390/ijms131216065

Jin, Y., Yang, H., Wei, Z., Ma, H., and Ge, X. (2013). Rice male development under drought stress: phenotypic changes and stage-dependent transcriptomic reprogramming. *Mol. Plant*. 6, 1630-1645. doi: 10.1093/mp/sst067

Kakumanu, A., Ambavaram, M. M., Klumas, C., Krishnan, A., Batlang, U., Myers, E., et al. (2012). Effects of drought on gene expression in maize reproductive and leaf meristem tissue revealed by RNA-Seq. *Plant Physiol*. 160, 846-867. doi: 10.1104/pp.112.200444

Katiyar, A., Smita, S., Muthusamy, S. K., Chinnusamy, V., Pandey, D. M., and Bansal, K. C. (2015). Identification of novel drought-responsive microRNAs and trans-acting siRNAs from *Sorghum bicolor* (L.) Moench by high-throughput sequencing analysis. *Front. Plant Sci.* 6, 506. doi: 10.3389/fpls.2015.00506

Katam, R., Sakata, K., Suravajhala, P., Pechan, T., Kambiranda, D. M., Naik, K. S., et al. (2016). Comparative leaf proteomics of drought-tolerant and -susceptible peanut in response to water stress. *J. Proteomics* 143, 209-226. doi: 10.1016/j.jprot.2016.05.031

Kausar, R., Arshad, M., Shahzad, A., and Komatsu, S. (2013). Proteomics analysis of sensitive and tolerant barley genotypes under drought stress. *Amino Acids* 44, 345-593. doi: 10.1007/s00726-012-1338-3

Ke, Y., Han, G., He, H., and Li, J. (2009). Differential regulation of proteins and phosphoproteins in rice under drought stress. *Biochem. Biophys. Res. Commun*. 379, 133-138. doi: 10.1016/j.bbrc.2008.12.067

Khan, M. N., and Komatsu, S. (2016). Proteomic analysis of soybean root including hypocotyl during recovery from drought stress. *J. Proteomics* 144, 39-50. doi: 10.1016/j.jprot.2016.06.006.

Koh, J., Chen, G., Yoo, M. J., Zhu, N., Dufresne, D., Erickson, J. E., et al. (2015). Comparative proteomic analysis of brassica napus in response to drought stress. *J. Proteome Res*. 14, 3068-3081. doi: 10.1021/pr501323d

Kottapalli, K. R., Zabet-Moghaddam, M., Rowland, D., Faircloth, W., Mirzaei, M., Haynes, P. A., et al. (2013). Shotgun label-free quantitative proteomics of water-deficit-stressed midmature peanut (*Arachis hypogaea* L.) seed. *J. Proteome Res*. 12, 5048-5057. doi: 10.1021/pr400936d

Krugman, T., Chagué, V., Peleg, Z., Balzergue, S., Just, J., Korol, A. B., et al. (2010). Multilevel regulation and signalling processes associated with adaptation to terminal drought in wild emmer wheat. *Funct. Integr. Genomic*. 10, 167-186. doi: 10.1007/s10142-010-0166-3

Krugman, T., Peleg, Z., Quansah, L., Chagué, V., Korol, A. B., Nevo, E., et al. (2011). Alteration in expression of hormone-related genes in wild emmer wheat roots associated with drought adaptation mechanisms. *Funct. Integr. Genomic*. 11, 565-583. doi: 10.1007/s10142-011-0231-6

Kurahashi, Y., Terashima, A., and Takumi, S. (2009). Variation in dehydration tolerance, ABA sensitivity and related gene expression patterns in D-genome progenitor and synthetic hexaploid wheat lines. *Int. J. Mol. Sci*. 10, 2733-2751. doi: 10.3390/ijms10062733

Kumar, D., Datta, R., Sinha, R., Ghosh, A., and Chattopadhyay, S. (2014). Proteomic profiling of γ-ECS overexpressed transgenic *Nicotiana* in response to drought stress. *Plant Signal Behav*. 9, e29246. doi: 10.4161/psb.29246

Kwasniewski, M., Daszkowska-Golec, A., Janiak, A., Chwialkowska, K., Nowakowska, U., Sablok, G., et al. (2016). Transcriptome analysis reveals the role of the root hairs as environmental sensors to maintain plant functions under water-deficiency conditions. *J. Exp. Bot.* 67, 1079-1094. doi: 10.1093/jxb/erv498

Kwon, S. W., Kim, M., Kim, H., and Lee, J. (2016). Shotgun quantitative proteomic analysis of proteins responding to drought stress in *Brassica rapa* L. (Inbred Line "Chiifu"). *Int. J. Genomics* 2016, 4235808. doi: 10.1155/2016/4235808

Le, D. T., Nishiyama, R., Watanabe, Y., Tanaka, M., Seki, M., Ham, le, H., et al. (2012). Differential gene expression in soybean leaf tissues at late developmental stages under drought stress revealed by genome-wide transcriptome analysis. *PLoS One* 7, e49522. doi: 10.1371/journal.pone.0049522

Lei, L., Shi, J., Chen, J., Zhang, M., Sun, S., Xie, S., et al. (2015). Ribosome profiling reveals dynamic translational landscape in maize seedlings under drought stress. *Plant J*. 84, 1206-1218. doi: 10.1111/tpj.13073

Lenka, S. K., Katiyar, A., Chinnusamy, V., and Bansal, K. C. (2011). Comparative analysis of drought-responsive transcriptome in Indica rice genotypes with contrasting drought tolerance. *Plant Biotechnol*. J. 9, 315-327. doi: 10.1111/j.1467-7652.2010.00560.x

Li, H., Dong, Y., Yin, H., Wang, N., Yang, J., Liu, X., et al. (2011). Characterization of the stress associated microRNAs in *Glycine max* by deep sequencing. [*BMC Plant Biol.*](https://www.ncbi.nlm.nih.gov/pubmed/?term=Characterization+of+the+stress+associated+microRNAs+in+Glycine+max+by+deep+sequencing) 11, 170. doi: 10.1186/1471-2229-11-170

Lima, J. M., Nath, M., Dokku, P., Raman, K. V., Kulkarni, K. P., Vishwakarma, C., et al. (2015). Physiological, anatomical and transcriptional alterations in a rice mutant leading to enhanced water stress tolerance. AoB *Plants* 7, plv023. doi: 10.1093/aobpla/plv023

Liu, H., Searle, I. R., Watson-Haigh, N. S., Baumann, U., Mather, D. E., Able, A. J., et al. (2015). Genome-wide identification of micrornas in leaves and the developing head of four durum genotypes during water deficit stress. *PLoS One* 10, e0142799. doi: 10.1371/journal.pone.0142799

Liu, Y., Zhou, M., Gao, Z., Ren, W, Yang, F., He, H., et al. (2015). RNA-seq analysis reveals MAPKKK family members related to drought tolerance in maize. *PLoS One* 10, e0143128. doi: 10.1371/journal.pone.0143128

Liu, Z., Xin, M., Qin, J., Peng, H., Ni, Z., Yao, Y., et al. (2015). Temporal transcriptome profiling reveals expression partitioning of homeologous genes contributing to heat and drought acclimation in wheat (*Triticum aestivum L.*). *BMC Plant Biol*. 15, 152. doi: 10.1186/s12870-015-0511-8

Lu, X., Chen, X., Mu, M., Wang, J., Wang, X., Wang, D., et al. (2016). Genome-wide analysis of long noncoding RNAs and their responses to drought stress in cotton (*Gossypiumhirsutum* L.). *PLoS One* 11, e0156723. doi: 10.1371/journal.pone.0156723

Lunardon, A., Forestan, C., Farinati, S., Axtell, M. J., and Varotto, S. (2016). Genome-wide characterization of maize small RNA loci and their regulation in the required to maintain repression6-1 (rmr6-1) mutant and long-term abiotic stresses. *Plant Physiol.* 170, 1535-1548. doi: 10.1104/pp.15.01205

Liu, J. X., and Bennett, J. (2011). Reversible and irreversible drought-induced changes in the anther proteome of rice (*Oryza sativa* L.) genotypes IR64 and Moroberekan. *Mol. Plant* 4, 59-69. doi: 10.1093/mp/ssq039

Liu, H., Sultan, M. A., Liu, X. L., Zhang, J., Yu, F., and Zhao, H. X. (2015). Physiological and comparative proteomic analysis reveals different drought responses in roots and leaves of drought-tolerant wild wheat (*Triticum boeoticum*). *PLoS One* 10, e0121852. doi: 10.1371/journal.pone.0121852

Luo, J., Tang, S., Peng, X., Yan, X., Zeng, X., Li, J., et al. (2015). Elucidation of cross-talk and specificity of early response mechanisms to salt and peg-simulated drought stresses in *Brassica napus* using comparative proteomic analysis. *PLoS One* 10, e0138974. doi: 10.1371/journal.pone.0138974

Marino, R., Ponnaiah, M., Krajewski, P., Frova, C., Gianfranceschi, L., Pè, M. E., et al. (2009). Addressing drought tolerance in maize by transcriptional profiling and mapping. *Mol. Genet Genomics*. 281, 163-179. doi: 10.1007/s00438-008-0401-y

Min, H., Chen, C., Wei, S., Shang, X., Sun, M., Xia, R., et al. (2016). Identification of drought tolerant mechanisms in maize seedlings based on transcriptome analysis of recombination inbred lines. *Front. Plant Sci*. 7, 1080. doi: 10.3389/fpls.2016.01080

Mirzaei, M., Soltani, N., Sarhadi, E., George, I. S., Neilson, K. A., Pascovici, D., et al. (2014). Manipulating root water supply elicits major shifts in the shoot proteome. *J. Proteome Res*. 13, 517-526. doi: 10.1021/pr400696u

Mirzaei, M., Pascovici, D., Atwell, B. J., and Haynes, P. A. (2012a). Differential regulation of aquaporins, small GTPases and V-ATPases proteins in rice leaves subjected to drought stress and recovery. *Proteomics* 12, 864-877. doi: 10.1002/pmic.201100389

Mirzaei, M., Soltani, N., Sarhadi, E., Pascovici, D., Keighley, T., Salekdeh, G. H., et al. (2012b). Shotgun proteomic analysis of long-distance drought signaling in rice roots. *J. Proteome Res*. 11, 348-358. doi: 10.1021/pr2008779

Mohammadi, P. P., Moieni, A., Hiraga, S., and Komatsu, S. (2012). Organ-specific proteomic analysis of drought-stressed soybean seedlings. *J. Proteomics* 75, 1906-1923. doi: 10.1016/j.jprot.2011.12.041

Mohammadi, P. P., Moieni, A., and Komatsu, S. (2012). Comparative proteome analysis of drought-sensitive and drought-tolerant rapeseed roots and their hybrid F1 line under drought stress. *Amino Acids* 43, 2137-2152. doi: 10.1007/s00726-012-1299-6

Moumeni, A., Satoh, K., Kondoh, H., Asano, T., Hosaka, A., Venuprasad, R., et al. (2011). Comparative analysis of root transcriptome profiles of two pairs of drought-tolerant and susceptible rice near-isogenic lines under different drought stress. *BMC Plant Biol*. 11, 174. doi: 10.1186/1471-2229-11-174

Moumeni, A., Satoh, K., Venuprasad, R., Serraj, R., Kumar, A., Leung, H., et al. (2015). Transcriptional profiling of the leaves of near-isogenic rice lines with contrasting drought tolerance at the reproductive stage in response to water deficit. *BMC Genomics* 16, 1110. doi: 10.1186/s12864-015-2335-1

Muthurajan, R., Shobbar, Z. S., Jagadish, S. V., Bruskiewich, R., Ismail, A., Leung, H., et al. (2011). Physiological and proteomic responses of rice peduncles to drought stress. *Mol. Biotechnol*. 48, 173-182. doi: 10.1007/s12033-010-9358-2

Nam, K. H., Shin, H. J., Pack, I. S., Park, J. H., Kim, H. B., and Kim, C. G. (2016). Metabolomic changes in grains of well-watered and drought-stressed transgenic rice. *J. Sci. Food Agric.* 96, 807-814. doi: 10.1002/jsfa.7152

Obata, T., Witt, S., Lisec, J., Palacios-Rojas, N., Florez-Sarasa, I., Yousfi, S., et al. (2015). Metabolite profiles of maize leaves in drought, heat, and combined stress field trials reveal the relationship between metabolism and grain yield. *Plant Physiol.* 169, 2665-2683. doi: 10.1104/pp.15.01164

Oh, M., and Komatsu, S. (2015). Characterization of proteins in soybean roots under flooding and drought stresses. *J. Proteomics* 114, 161-181. doi: 10.1016/j.jprot.2014.11.008

Opitz, N., Marcon, C., Paschold, A., Malik, W. A., Lithio, A., Brandt, R., et al. (2016). Extensive tissue-specific transcriptomic plasticity in maize primary roots upon water deficit. *J. Exp. Bot*. 67, 1095-1107. doi: 10.1093/jxb/erv453

Opitz, N., Paschold, A., Marcon, C., Malik, W. A., Lanz, C., Piepho, H. P., et al. (2014). Transcriptomic complexity in young maize primary roots in response to low water potentials. *BMC Genomics* 15, 741. doi: 10.1186/1471-2164-15-741

Padmalatha, K. V., Dhandapani, G., Kanakachari, M., Kumar, S., Dass, A., Patil, D. P., et al. (2012). Genome-wide transcriptomic analysis of cotton under drought stress reveal significant down-regulation of genes and pathways involved in fibre elongation and up-regulation of defense responsive genes. *Plant Mol. Biol.* 78, 223-246. doi: 10.1007/s11103-011-9857-y

Pasini, L., Bergonti, M., Fracasso, A., Marocco, A., and Amaducci, S. (2014). Microarray analysis of differentially expressed mRNAs and miRNAs in young leaves of sorghum under dry-down conditions. *J. Plant Physiol.*171, 537-548. doi: 10.1016/j.jplph.2013.12.014

Park, W., Scheffler, B. E., Bauer, P. J., and Campbell, B. T. (2012). Comparative transcriptomic analysis of roots of contrasting *Gossypium herbaceum* genotypes revealing adaptation to drought. [*BMC Plant Biol.*](https://www.ncbi.nlm.nih.gov/pubmed/?term=Genome-wide+identification+of+differentially+expressed+genes+under+water+deficit+stress+in+upland+cotton+(Gossypium+hirsutum+L.)) 12, 90. doi: 10.1186/1471-2229-12-90

Payton, P., Kottapalli, K. R., Kebede, H., Mahan, J. R., Wright, R. J., and Allen, R. D. (2011). Examining the drought stress transcriptome in cotton leaf and root tissue. *Biotechnol. Lett.* 33, 821-8.doi: 10.1007/s10529-010-0499-y

Paul, S., Gayen, D., Datta, S. K., and Datta, K. (2015). Dissecting root proteome of transgenic rice cultivars unravels metabolic alterations and accumulation of novel stress responsive proteins under drought stress. *Plant Sci*. 234, 133-143. doi: 10.1016/j.plantsci.2015.02.006

Peremarti, A., Marè, C., Aprile, A., Roncaglia, E., Cattivelli, L., Villegas, D., et al. (2014). Transcriptomic and proteomic analyses of a pale-green durum wheat mutant shows variations in photosystem components and metabolic deficiencies under drought stress. *BMC Genomics* 15, 125. doi: 10.1186/1471-2164-15-125

Peng, Z., Wang, M., Li, F., Lv, H., Li, C., and Xia, G. (2009). A proteomic study of the response to salinity and drought stress in an introgression strain of bread wheat. *Mol. Cell Proteomics* 8, 2676-2686. doi: 10.1074/mcp.M900052-MCP200

Prince, S. J., Joshi, T., Mutava, R. N., Syed, N., Joao VitorMdos, S., Patil, G., et al. (2015). Comparative analysis of the drought-responsive transcriptome in soybean lines contrasting for canopy wilting. [*Plant Sci.*](https://www.ncbi.nlm.nih.gov/pubmed/?term=Comparative+analysis+of+the+drought-responsive+transcriptome+insoybean+lines+contrasting+for+canopy+wilting) 240, 65-78. doi: 10.1016/j.plantsci.2015.08.017

Qin, Y., Song, W., Xiao, S., Yin, G., Zhu, Y., Yan, Y., et al. (2014). Stress-related genes distinctly expressed in unfertilized wheat ovaries under both normal and water deficit conditions whereas differed in fertilized ovaries. *J. Proteomics* 102, 11-27. doi: 10.1016/j.jprot.2014.02.028

Qin, N., Xu, W., Hu, L., Li, Y., Wang, H., Qi, X., et al. (2015). Drought tolerance and proteomics studies of transgenic wheat containing the maize C4 phosphoenolpyruvate carboxylase (*PEPC*) gene. *Protoplasma* doi:10.1007/s00709-015-0906-2

Rabara, R. C., Tripathi, P., Choudhary, M. K., Timko, M. P., Shen, Q. J., and Rushton, P. J. (2015a). Transcriptome profiling of tobacco under water deficit conditions. *Genom. Data* 5, 61-63. doi: 10.1016/j.gdata.2015.05.025

Rabara, R. C., Tripathi, P., Reese, R. N., Rushton, D. L., Alexander, D., Timko, M. P., et al. (2015b). Tobacco drought stress responses reveal new targets for *Solanaceae* crop improvement. *BMC Genomics* 16, 484.doi: 10.1186/s12864-015-1575-4

Rabello, F. R., Villeth, G. R., Rabello, A. R., Rangel, P. H., Guimarães, C. M., Huergo, L. F., et al. (2014). Proteomic analysis of upland rice (*Oryza sativa* L.) exposed to intermittent water deficit. *Protein J*. 33, 221-230. doi: 10.1007/s10930-014-9554-1

Rabello, A. R., Guimarães, C. M., Rangel, P. H., da Silva, F. R., Seixas, D., de Souza, E., et al. (2008). Identification of drought-responsive genes in roots of upland rice (*Oryza sativa* L). *BMC Genomics* 9, 485. doi: 10.1186/1471-2164-9-485

Ranjan, A., Nigam, D., Asif, M. H., Singh, R., Ranjan, S., Mantri, S., et al. (2012). Genome wide expression profiling of two accession of *G. herbaceum* L. in response to drought. *BMC Genomics* 13, 94.doi: 10.1186/1471-2164-13-94

Ranjan, A., [Pandey, N](https://www.ncbi.nlm.nih.gov/pubmed/?term=Pandey N%5BAuthor%5D&cauthor=true&cauthor_uid=23194183)., [Lakhwani, D](https://www.ncbi.nlm.nih.gov/pubmed/?term=Lakhwani D%5BAuthor%5D&cauthor=true&cauthor_uid=23194183)., [Dubey, N. K](https://www.ncbi.nlm.nih.gov/pubmed/?term=Dubey NK%5BAuthor%5D&cauthor=true&cauthor_uid=23194183)., [Pathre, U. V](https://www.ncbi.nlm.nih.gov/pubmed/?term=Pathre UV%5BAuthor%5D&cauthor=true&cauthor_uid=23194183)., and [Sawant, S. V](https://www.ncbi.nlm.nih.gov/pubmed/?term=Sawant SV%5BAuthor%5D&cauthor=true&cauthor_uid=23194183). (2012). Comparative transcriptomic analysis of roots of contrasting *Gossypium herbaceum* genotypes revealing adaptation to drought. *BMC Genomics* 13, 680.doi: 10.1186/1471-2164-13-680

Raorane, M. L., Pabuayon, I. M., Miro, B., Kalladan, R., Reza-Hajirezai, M., Oane, R. H., et al. (2015). Variation in primary metabolites in parental and near-isogenic lines of the QTL qDTY12.1 : altered roots and flag leaves but similar spikelets of rice under drought. *Mol. Breeding.* 35, 138. doi: 10.1007/s11032-015-0322-5

Reddy, S. K., Liu, S., Rudd, J. C., Xue, Q., Payton, P., Finlayson, S. A., et al. (2014). Physiology and transcriptomics of water-deficit stress responses in wheat cultivars TAM 111 and TAM 112. *J. Plant Physiol*. 171, 1289-1298. doi: 10.1016/j.jplph.2014.05.005

Rodrigues, F. A., Marcolino-Gomes, J., de FátimaCorrêaCarvalho, J., do Nascimento, L. C., Neumaier, N., Farias, J. R., et al. (2012). Subtractive libraries for prospecting differentially expressed genes in the soybean under water deficit. *Genet. Mol. Biol.* 35, 304-314. doi: 10.1590/S1415-47572012000200011

Rodrigues, F. A., Fuganti-Pagliarini, R., Marcolino-Gomes, J., Nakayama, T. J., Molinari, H. B., Lobo, F. P., et al. (2015). Daytime soybean transcriptome fluctuations during water deficit stress. *BMC Genomics* 16, 505.doi: 10.1186/s12864-015-1731-x

Rollins, J. A., Habte, E., Templer, S. E., Colby, T., Schmidt, J., and von Korff, M. (2013). Leaf proteome alterations in the context of physiological and morphological responses to drought and heat stress in barley (*Hordeum vulgare* L.). *J. Exp. Bot*. 64, 3201-3212. doi: 10.1093/jxb/ert158

Salekdeh, G. H., Siopongco, J., Wade, L. J., Ghareyazie, B., and Bennett, J. (2002). Proteomic analysis of rice leaves during drought stress and recovery. *Proteomics* 2, 1131-1145

Senakoon, W., Nuchadomrong, S., Chiou, R. Y., Senawong, G., Jogloy, S., Songsri, P., et al. (2015). Identification of peanut seed prolamins with an antifungal role by 2D-GE and drought treatment. *Biosci. Biotechnol Biochem*. 79, 1771-1778. doi: 10.1080/09168451.2015.1056508

Sharma, V., Sekhwal, M. K., Swami, A. K., and Sarin, R. (2012). Identification of drought responsive proteins using gene ontology hierarchy. *Bioinformation* 8, 595-599. doi: 10.6026/97320630008595

[Shin, J. H](https://www.ncbi.nlm.nih.gov/pubmed/?term=Shin JH%5BAuthor%5D&cauthor=true&cauthor_uid=25644024)., [Vaughn, J. N](https://www.ncbi.nlm.nih.gov/pubmed/?term=Vaughn JN%5BAuthor%5D&cauthor=true&cauthor_uid=25644024)., [Abdel-Haleem, H](https://www.ncbi.nlm.nih.gov/pubmed/?term=Abdel-Haleem H%5BAuthor%5D&cauthor=true&cauthor_uid=25644024)., [Chavarro, C](https://www.ncbi.nlm.nih.gov/pubmed/?term=Chavarro C%5BAuthor%5D&cauthor=true&cauthor_uid=25644024)., [Abernathy, B](https://www.ncbi.nlm.nih.gov/pubmed/?term=Abernathy B%5BAuthor%5D&cauthor=true&cauthor_uid=25644024)., [Kim, K. D](https://www.ncbi.nlm.nih.gov/pubmed/?term=Kim KD%5BAuthor%5D&cauthor=true&cauthor_uid=25644024)., et al. (2015). Transcriptomic changes due to water deficit define a general soybean response and accession-specific pathways for drought avoidance. *BMC Plant Biol.* 15, 26. doi: 10.1186/s12870-015-0422-8

Sicher, R. C., and Barnaby, J. Y. (2012). Impact of carbon dioxide enrichment on the responses of maize leaf transcripts and metabolites to water stress. *Physiol. Plant.* 144, 238-253. doi: 10.1111/j.1399-3054.2011.01555.x

Singh, R., [Pandey, N](https://www.ncbi.nlm.nih.gov/pubmed/?term=Pandey N%5BAuthor%5D&cauthor=true&cauthor_uid=25802007)., Kumar, A., and Shirke, P. A. (2016). Physiological performance and differential expression profiling of genes associated with drought tolerance in root tissue of four contrasting varieties of two *Gossypium* species. *Protoplasma* 253, 163-174. doi: 10.1007/s00709-015-0800-y

Singh, R., Pandey, N., Naskar, J., and Shirke, P. A. (2015). Physiological performance and differential expression profiling of genes associated with drought tolerance in contrasting varieties of two *Gossypium* species. *Protoplasma* 252, 423-438. doi: 10.1007/s00709-014-0686-0

Song, L., Prince, S., Valliyodan, B., Joshi, T., Maldonado dos Santos, J. V., Wang, J., et al. (2016). Genome-wide transcriptome analysis of soybean primary root under varying water-deficit conditions. *BMC Genomics* 17, 57.doi: 10.1186/s12864-016-2378-y

Shu, L., Lou, Q., Ma, C., Ding, W., Zhou, J., Wu, J., et al. (2011). Genetic, proteomic and metabolic analysis of the regulation of energy storage in rice seedlings in response to drought. *Proteomics* 11, 4122-4138. doi: 10.1002/pmic.201000485

Tavakol, E., Sardaro, M. L., Shariati, J. V., Rossini, L., and Porceddu, E. (2014). Isolation, promoter analysis and expression profile of Dreb2 in response to drought stress in wheat ancestors. *Gene*. 549, 24-32. doi: 10.1016/j.gene.2014.07.020

Thatcher, S. R., Danilevskaya, O. N., Meng, X., Beatty, M., Zastrow-Hayes, G., and Harris, C. (2016). Genome-wide analysis of alternative splicing during development and drought stress in maize. *Plant Physiol*. 170, 586-599. doi: 10.1104/pp.15.01267

Tian, X. J., Long, Y., Wang, J., Zhang, J. W., Wang, Y. Y., Li, W. M., et al. (2015). De novo transcriptome assembly of common wild rice (*Oryza Rufipogon Griff.*) and discovery of drought-response genes in root tissue based on transcriptomic data. *PLoS One* 10, e0131455. doi: 10.1371/journal.pone.0131455

Tripathi, P., Rabara, R. C., Shen, Q. J., and Rushton, P. J. (2015). Transcriptomics analyses of soybean leaf and root samples during water-deficit. *Genom. Data* 5, 164-166. doi: 10.1016/j.gdata.2015.05.036

Vincent, D., Lapierre, C., Pollet, B., Cornic, G., Negroni, L., and Zivy, M. (2005). Water deficits affect caffeate O-methyltransferase, lignification, and related enzymes in maize leaves. A proteomic investigation. *Plant Physiol*. 137, 949-960. doi: 10.1104/pp.104.050815

Vítámvás, P., Urban, M. O., Škodáček, Z., Kosová, K., Pitelková, I., Vítámvás, J., et al. (2015). Quantitative analysis of proteome extracted from barley crowns grown under different drought conditions. *Front. Plant Sci*. 6, 479. doi: 10.3389/fpls.2015.00479

[Vojta, P](https://www.ncbi.nlm.nih.gov/pubmed/?term=Vojta P%5BAuthor%5D&cauthor=true&cauthor_uid=26877151)., [Kokáš, F](https://www.ncbi.nlm.nih.gov/pubmed/?term=Kokáš F%5BAuthor%5D&cauthor=true&cauthor_uid=26877151)., [Husičková, A](https://www.ncbi.nlm.nih.gov/pubmed/?term=Husičková A%5BAuthor%5D&cauthor=true&cauthor_uid=26877151)., [Grúz, J](https://www.ncbi.nlm.nih.gov/pubmed/?term=Grúz J%5BAuthor%5D&cauthor=true&cauthor_uid=26877151)., [Bergougnoux, V](https://www.ncbi.nlm.nih.gov/pubmed/?term=Bergougnoux V%5BAuthor%5D&cauthor=true&cauthor_uid=26877151)., [Marchetti, C. F](https://www.ncbi.nlm.nih.gov/pubmed/?term=Marchetti CF%5BAuthor%5D&cauthor=true&cauthor_uid=26877151)., et al. (2016). Whole transcriptome analysis of transgenic barley with altered cytokinin homeostasis and increased tolerance to drought stress. [*N. Biotechnol*](https://www.ncbi.nlm.nih.gov/pubmed/?term=Whole+transcriptome+analysis+of+transgenic+barley+with+altered+cytokinin+homeostasis+and+increased+tolerance+to+drought+stress). 33, 676-691. doi: 10.1016/j.nbt.2016.01.010

Wang, D., Pan, Y., Zhao, X., Zhu, L., Fu, B., and Li, Z. (2011). Genome-wide temporal-spatial gene expression profiling of drought responsiveness in rice. *BMC Genomics* 12, 149. doi: 10.1186/1471-2164-12-149

Wang, N., Zhao, J., He, X., Sun, H., Zhang, G., and Wu, F. (2015). Comparative proteomic analysis of drought tolerance in the two contrasting Tibetan wild genotypes and cultivated genotype. *BMC Genomics* 16, 432. doi: 10.1186/s12864-015-1657-3

Wang, X., and Komatsu, S. (2016). Gel-Free/Label-Free proteomic analysis of endoplasmic reticulum proteins in soybean root tips under flooding and drought stresses. *J. Proteome Res*. 15, 2211-2227. doi: 10.1021/acs.jproteome.6b00190

Wang, X., Oh, M., Sakata, K., and Komatsu, S. (2016). Gel-free/label-free proteomic analysis of root tip of soybean over time under flooding and drought stresses. *J. Proteomics* 130, 42-55. doi: 10.1016/j.jprot.2015.09.007

Wang, X., Vignjevic, M., Jiang, D., Jacobsen, S., and Wollenweber, B. (2014). Improved tolerance to drought stress after anthesis due to priming before anthesis in wheat (*Triticum aestivum* L.) var. Vinjett. *J. Exp. Bot*. 65, 6441-6456. doi: 10.1093/jxb/eru362

Wendelboe-Nelson, C., and Morris, P. C. (2012). Proteins linked to drought tolerance revealed by DIGE analysis of drought resistant and susceptible barley varieties. *Proteomics* 12, 3374-3385. doi: 10.1002/pmic.201200154

Wu, Y., Mirzaei, M., Pascovici, D., Chick, J. M., Atwell, B. J., and Haynes, P. A. (2016). Quantitative proteomic analysis of two different rice varieties reveals that drought tolerance is correlated with reduced abundance of photosynthetic machinery and increased abundance of ClpD1 protease. *J. Proteomics* 143, 73-82. doi: 10.1016/j.jprot.2016.05.014

Xie, H., Yang, D. H., Yao, H., Bai, G., Zhang, Y. H., and Xiao, B. G. (2016). iTRAQ-based quantitative proteomic analysis reveals proteomic changes in leaves of cultivated tobacco (*Nicotiana tabacum*) in response to drought stress. *Biochem. Biophys. Res. Commun*. 469, 768-775. doi: 10.1016/j.bbrc.2015.11.133

Yang, L., Jiang, T., Fountain, J. C., Scully, B. T., Lee, R. D., Kemerait, R. C., et al. (2014). Protein profiles reveal diverse responsive signaling pathways in kernels of two maize inbred lines with contrasting drought sensitivity. *Int. J. Mol. Sci*. 15, 18892-18918. doi: 10.3390/ijms151018892

Yang, F., Jørgensen, A. D., Li, H., Søndergaard, I., Finnie, C., Svensson, B., et al. (2011). Implications of high-temperature events and water deficits on protein profiles in wheat (*Triticum aestivum* L. cv. Vinjett) grain. *Proteomics* 11, 1684-1695. doi: 10.1002/pmic.201000654

Yue, G., Zhuang, Y., Li, Z., Sun, L., and Zhang, J. (2008). Differential gene expression analysis of maize leaf at heading stage in response to water-deficit stress. *Bioscience Rep*. 28, 125-134. doi: 10.1042/BSR20070023

Zeng, X., Bai, L., Wei, Z., Yuan, H., Wang, Y., Xu, Q., et al. (2016). Transcriptome analysis revealed the drought-responsive genes in Tibetan hulless barley. [*BMC Genomics*](https://www.ncbi.nlm.nih.gov/pubmed/?term=Transcriptome+analysis+revealed+the+drought-responsive+genes+in+Tibetan+hulless+barley) 17, 386.doi: 10.1186/s12864-016-2685-3

Zhang, H., Ni, Z., Chen, Q., Guo, Z., Gao, W., Su, X., et al. (2016). Proteomic responses of drought-tolerant and drought-sensitive cotton varieties to drought stress. *Mol. Genet. Genomics* 291, 1293-1303. doi: 10.1007/s00438-016-1188-x

Zhang, M., Lv, D., Ge, P., Bian, Y., Chen, G., Zhu, G., et al. (2014). Phosphoproteome analysis reveals new drought response and defense mechanisms of seedling leaves in bread wheat (*Triticum aestivum* L.). *J. Proteomics* 109, 290-308. doi: 10.1016/j.jprot.2014.07.010

Zhang, Y. P., E, Z. G., Jiang, H., Wang, L., Zhou, J., and Zhu, D. F. (2015). A comparative study of stress-related gene expression under single stress and intercross stress in rice. *Genet. Mol. Res*. 14, 3702-3717. doi: 10.4238/2015.April.17.20

Zhang, Z. F., Li, Y. Y., and Xiao, B. Z. (2016). Comparative transcriptome analysis highlights the crucial roles of photosynthetic system in drought stress adaptation in upland rice. *Sci. Rep*. 6, 19349. doi: 10.1038/srep19349

Zheng, M., Meng, Y., Yang, C., Zhou, Z., Wang, Y., and Chen, B. (2014). Protein expression changes during cotton fiber elongation in response to drought stress and recovery. *Proteomics* 14, 1776-1795. doi: 10.1002/pmic.201300123

Zheng, J., Fu, J., Gou, M., Huai, J., Liu, Y., Jian, M., et al. (2010). Genome-wide transcriptome analysis of two maize inbred lines under drought stress. *Plant Mol. Biol*. 72, 407-421. doi: 10.1007/s11103-009-9579-6

Zhu, Y. N., [Shi, D. Q](https://www.ncbi.nlm.nih.gov/pubmed/?term=Shi DQ%5BAuthor%5D&cauthor=true&cauthor_uid=24224045)., [Ruan, M. B](https://www.ncbi.nlm.nih.gov/pubmed/?term=Ruan MB%5BAuthor%5D&cauthor=true&cauthor_uid=24224045)., [Zhang, L. L](https://www.ncbi.nlm.nih.gov/pubmed/?term=Zhang LL%5BAuthor%5D&cauthor=true&cauthor_uid=24224045)., [Meng, Z. H](https://www.ncbi.nlm.nih.gov/pubmed/?term=Meng ZH%5BAuthor%5D&cauthor=true&cauthor_uid=24224045)., [Liu, J](https://www.ncbi.nlm.nih.gov/pubmed/?term=Liu J%5BAuthor%5D&cauthor=true&cauthor_uid=24224045)., et al. (2013). Transcriptome analysis reveals crosstalk of responsive genes to multiple abiotic stresses in cotton (*Gossypiumhirsutum* L.). [*PLoS One*](https://www.ncbi.nlm.nih.gov/pubmed/?term=Transcriptome+Analysis+Reveals+Crosstalk+of+Responsive+Genes+to+Multiple+Abiotic+Stresses+in+Cotton+(Gossypium+hirsutum+L.)) 8, e80218. doi: 10.1371/journal.pone.0080218
